# Melatonin and Hippo Pathway: Is There Existing Cross-Talk?

**DOI:** 10.3390/ijms18091913

**Published:** 2017-09-06

**Authors:** Federica Lo Sardo, Paola Muti, Giovanni Blandino, Sabrina Strano

**Affiliations:** 1 Oncogenomic and Epigenetic Unit, Molecular Chemoprevention Group, Department of Research, Diagnosis and Innovative Technologies, Translational Research Area, Regina Elena National Cancer Institute, via Elio Chianesi 53, 00144 Rome, Italy; federica.losardo@ifo.gov.it (F.L.S.); giovanni.blandino@ifo.gov.it (G.B.); 2 Department of Oncology, Juravinski Cancer Center, McMaster University, Hamilton, ON L8S 4L8, Canada; muti@mcmaster.ca

**Keywords:** melatonin, cancer, melatonin receptors, GPCR signaling, Hippo pathway

## Abstract

Melatonin is an indolic hormone that regulates a plethora of functions ranging from the regulation of circadian rhythms and antioxidant properties to the induction and maintenance of tumor suppressor pathways. It binds to specific receptors as well as to some cytosolic proteins, leading to several cellular signaling cascades. Recently, the involvement of melatonin in cancer insurgence and progression has clearly been demonstrated. In this review, we will first describe the structure and functions of melatonin and its receptors, and then discuss both molecular and epidemiological evidence on melatonin anticancer effects. Finally, we will shed light on potential cross-talk between melatonin signaling and the Hippo signaling pathway, along with the possible implications for cancer therapy.

## 1. Introduction

Melatonin (*N*-acetyl-5-methoxy tryptamine) is a pleiotropic neurohormone mainly secreted by the pineal gland and partially by other peripheral organs that are widely distributed, including in the gut, gonads, retina, and immune-competent cells [1]. Its production is tightly regulated by light/dark signals coming from the retina, following a circadian rhythm, with a peak during the night and relatively lower concentrations during the day, when the light turns off its production [2,3,4]. Melatonin peak levels are higher in new-borns and start to decline in the elderly [5].

The physiological input for pineal melatonin production starts in a subgroup of Retinal Ganglion Cells sensitive to a specific light-blue wavelength [2,3] that transmits information to the pineal gland through the retino-hypotalamic tract and induces the transcription and stabilization of *N*-acetyltransferase (NAT) enzyme [6,7]. This latter, together with the hydroxyindole-*O*-methyltransferase enzyme (HIOMT), is responsible for converting serotonin to melatonin [3,8,9]. During the day, light inhibits NAT and melatonin production [10].

Once produced, melatonin is released into the cerebrospinal fluid and capillaries and reaches all the body’s tissues, with concentrations between picomolars and nanomolars. It is metabolized by the liver and secreted in the urine, where its major metabolite, 6-sulfatoxy-melatonin (aMT6s), correlates with melatonin’s nocturnal plasma peaks [11,12,13]. Chronic exposure to artificial light at night deregulates melatonin levels, as shown in rodent models and in cohort studies of night-shift workers [14]. In addition, an inverse correlation between melatonin levels and tumor incidence has been reported in prospective nested case control studies [15,16,17,18,19,20,21,22], suggesting that supplementation with melatonin might be proposed as cancer chemopreventive treatment in human clinical studies [23,24,25].

## 2. Melatonin Membrane Receptors

Melatonin controls a plethora of physiological processes including regulation of sleep-wake rhythm, temperature and physiologic activities in the circadian clock, blood pressure regulation, stimulation of bone metabolism, immune function, reproductive functions, memory formation, cell differentiation and proliferation, inhibition of oxidative stress and inflammation processes [26,27,28,29,30,31,32,33,34,35,36]. All these functions employ both receptor-dependent and receptor-independent mechanisms. The two main membrane receptors, MTNR1A and MTRN1B, also known as MT1 and MT2, belong to the superfamily of G-protein coupled receptors (GPCRs), which constitute the largest family of membrane receptors with approximately 1000 members and respond to a wide variety of extracellular stimuli (hormones, neurotransmitters, or growth factors) controlling physiological processes such as cellular metabolism, secretion, cell differentiation, and growth [37]. MT1 and MT2 exist in both homo-dimeric and hetero-dimeric forms, and share high sequence homology [38]. They are expressed in several areas of the central nervous system (CNS), in the retina, the gastro-intestinal tract, arteries and immune cells [33]. They show different affinities for melatonin: MT2 has a 5-fold higher affinity than MT1, in both humans and other species [38,39,40]. MT3, a low affinity binding receptor, is a Quinone reductase 2, an enzyme that catalyzes the reduction of quinones into quinols having important implications on oxidative stress [41,42].

### MT1 and MT2 Mediated Signal Transduction

Upon agonist binding, cytoplasmic heterotrimeric G proteins that are comprised of Gα, β and γ subunits dissociate from GPCR transmembrane receptors [43]. The 15 different Gα subunits are classified into four families, Gα12/13, Gαq/11, Gαi/o, and Gαs [44], which in turn act on individual effectors such as adenylate cyclase (AC), phosphodiesterase (PDE), phospholipase C (PLC), or ion channels to affect the levels of associated second messengers including 3′,5′-cyclic adenosine or guanosine monophosphate (cAMP and cGMP), inositol triphosphate (IP_3_), and calcium [45].

MT1 and MT2 receptors mainly associate with Gαi proteins and to a lesser extent with Gαq/11 and Gαs proteins [46,47,48,49,50,51,52,53,54,55] which can couple to multiple signal transduction cascades, either alternately, or concomitantly in the same tissue [56]. In general, the signal transduction pathways induced by melatonin receptors are cell type and tissue specific [33] leading to unique cellular responses and suggesting a potential crosstalk with other signaling pathways. For example, MT1 receptor activation by melatonin may lead to different and in some cases opposite signaling pathways, depending on which Gα protein is activated. In general, Gαi activation leads to the inhibition of the adenyl cyclase activity with consequent inhibition of cyclic AMP (cAMP) formation, inhibition of protein kinase A (PKA) activity, and reduced phosphorylation and transcriptional activity of the cAMP-responsive element binding (CREB) as well as activation of phospholipase C β [48,57,58,59,60,61,62,63,64]. Conversely, in other systems such as Cos7 fibroblasts, HEK293 and MCF7 cells, activation of Gαq and Gαs proteins coupled to MT1 receptors leads to an increase of cAMP formation. Increased intracellular cAMP in turn activates PKA and PKC, which causes the inhibition of NF-ΚB (Nuclear Factor Kappa-light-chain-enhancer of activated B cells) with consequent derepression of the oncosuppressor p27^kip1^ and attenuation of the androgen response in prostate cells [53,54,55,65,66,67], activation of JNK in Cos7 cells [68], and phosphorylation of ERK1/2 in HEK 293 cells [69].

## 3. Melatonin and Nuclear Receptors: Contrasting Evidence

In the 90’s and a few years later, some studies showed that melatonin and its analogues can bind in vitro to nuclear receptors belonging to the family of retinoid Z receptor/Retinoid Orphan Receptor alpha (RZR/RORα) [70,71,72,73,74]. These receptors are organized into the following structural domains: a N-terminal transactivating domain, a DNA-binding domain, a variable domain, and a ligand-binding domain [75]. Once activated, they bind ROR response elements (ROREs) on the chromatin (TAAA/TNTA*GGTCA* motif) primarily as monomers [76,77,78,79] and regulate genes involved in cell differentiation, immune response, lipid metabolism, CNS development, tumour growth and inflammation [33,71,72,73,80,81,82,83,84,85,86]. Biologically, a role for melatonin in the downregulation of 5-lipooxygenase gene through RZR/RORα receptors has been shown by Carlsberg’s group in B lymphocytes [87]. However, Carlberg’s group in 1997 retracted the report that melatonin is a direct ligand of these nuclear receptors because they could not reproduce their results. Yet, the above mentioned study suggested a positive regulation of RZR/RORα by melatonin either in transcriptional activation or in repression of target genes [70,71,72,73,87], even if a direct binding of melatonin to receptors is arguable. In 2011, the group of Lardone and coworkers showed a direct interaction between melatonin and nuclear receptors in T lymphocytes and a negative regulation of nuclear RORα levels by melatonin. Other works showed a possible negative regulation of RZR/RORα by melatonin in different experimental systems [88,89], while in models of gastric cancer melatonin has been shown to negatively regulate RZR/RORγ [90].

## 4. Oncoprotective Role of Melatonin: In Vitro Evidence

Epidemiological studies have suggested that melatonin decreases the risk of developing different types of cancer. Recently, the molecular mechanism by which melatonin exerts its anticancer effects has been revised [91,92]. This might occur either through downregulation of oncogenic pathways or via activation of tumor suppressor activities.

**Figure 1:**
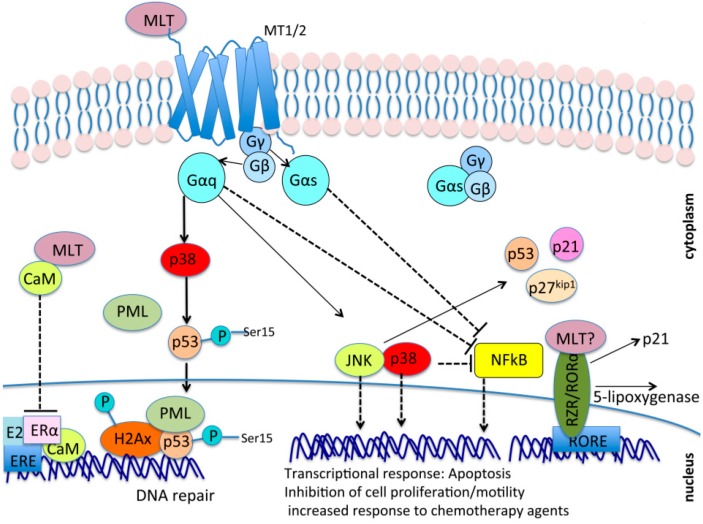
Oncosuppressive mechanisms mediated by melatonin. Melatonin (MLT) signaling has been shown to reduce the abundance and transcriptional activity of the NF-κB (nuclear factor kappa-light-chain-enhancer of activated B cells) transcription factor as well as to activate phosphorylation cascades mediated by mitogen-activated protein kinases (MAPKs) such as MEK1/2, ERK1/2, JNK, and p38. Both NF-κB inhibition and MAPKs activation in turn inhibit cell growth and motility, and promote apoptosis and DNA damage repair through mechanisms involving the accumulation of oncosuppressors such as p53, p27^kip1^, and p21, activation of DNA repair complexes such as P53/PML/H2AX on DNA damage sites, and transcriptional control of genes involved in the cell cycle, apoptosis, and invasiveness. Even though it is still a matter of debate, there is the possibility that melatonin can also bind to nuclear receptors RZR/ROR, controlling the transcription of RORE (ROR response Elements) on genes of the retinoic acid response, among which are several genes controlling cell cycle progression and cell growth (p21, 5-lipoxygenase, and others). Finally, melatonin can bind to the intracellular protein calmodulin (CaM) and reduce the Estrogen Receptor α (ERα) response in ER positive cells by impairing the formation of a proper E2–ERα–CaM complex on Estrogen Receptor Elements (EREs) on target genes. Arrows indicate activation, while dashed and blunt lines indicate inhibition. Activation indicates an increase in protein or activity levels, while inhibition indicates a decrease in protein or activity levels.

Melatonin can activate phosphorylation cascades, mediated by MEK1/2, ERK1/2, JNK and p38 MAPK, through binding to its membrane receptors MT1 and MT2 [93,94,95,96,97]. In particular, our group and others have shown that the binding of melatonin to MT1 and MT2 triggers a phosphorylation cascade, mediated in part by p38, which leads to the activation of p53 through phosphorylation of Ser15. This leads to a transient cell cycle arrest through the accumulation of tumor suppressive proteins (see Figure 1) and the induction of DNA repair mechanisms that prevent the accumulation of DNA mutations in response to DNA damage induced by chemotherapeutic agents or ionizing radiations. These events occurred both in normal and tumor cells only in the presence of intact MT1 and MT2 signaling [98,99,100].

Melatonin also causes the reduction of the abundance and the transcriptional activity of the NF-κB (nuclear factor kappa-light-chain-enhancer of activated B cells) transcription factor, leading to reduced proliferation and metastasis as well as increased apoptosis in basal conditions or in response to chemotherapeutic agents in several models of cancer including breast cancer, prostate cancer, colon and gastric cancer, pancreatic cancer, renal carcinoma, and hepatoma [55,93,95,101,102,103,104]. Part of the inhibition of the NF-κB transcriptional activity is elicited through the activation of JNK and p38 [93]. Oncosuppressive pathways induced by melatonin were also observed in vitro in glioblastoma [105,106] and osteosarcoma [107]. It is important to mention that beside its effects on cancer cells, melatonin protects from apoptosis normal cells like spermatozoa [108], cells in the liver [109], in the nervous [110] and immune systems [84,111,112,113,114,115,116,117,118,119,120], in which melatonin counteracts aging-related diseases, and stimulates immune cells activation and proliferation, respectively. Melatonin increases the number of effector T cells and decreases the number of regulative T cells (Tregs) [119,120]. Tregs have an inhibitory effect on anti-cancer immunity and some tumor cells are able to upregulate and recruit Tregs to escape the antitumor effect of the cellular immune system [121].

In summary, melatonin promotes apoptosis in certain circumstances, for example in cancer cells and in Treg cells, while it protects normal cells from apoptosis, including cells of the immune system that actively counteract infections and tumors.

The net effect of these opposite mechanisms is the protection of the whole organism from inflammation, aging-related diseases, cancer development, and progression.

Melatonin also inhibits cancer cell migration and invasiveness by increasing the expression of cell adhesion molecules [122,123,124,125] and by reducing the expression of the RhoA kinase ROCK involved in progression and metastasization of several tumors [124,126,127].

Moreover, melatonin has been shown to inhibit the expression of stemness-related genes, [105,128,129] to inhibit stemness-related pathways [105,106] to improve the response to several anticancer therapies [102,103,130,131,132,133,134], and to inhibit angiogenesis [127,135,136,137,138,139,140,141,142,143,144,145,146]. Finally, in Androgen Receptor (AR) and Estrogen Receptor (ER) positive cells, melatonin inhibits the AR [54,65,147,148,149] and ER response [128,150,151,152,153] through different mechanisms either mediated by MT1 or independent of MT1 receptor binding.

In general, MT1 seems to play a prominent role in triggering anti-tumor cellular responses mediated by melatonin [53,54,55,154,155,156,157,158,159], although in some experimental models MT2 has been shown to be required too [160,161].

Melatonin can also counteract tumor formation through mechanisms independent of MT1 and MT2. For example, through calmodulin (CaM) binding, melatonin interferes with the transcription of Estrogen Receptor α (ERα) genes in response to estrogen (E_2_). The formation of a melatonin–CaM complex, in fact, impairs the formation of a proper E_2_–ERα–CaM complex on ERα targets [153]. Moreover, in models of colon cancer, gastric cancer [82,90,162,163], and ovarian carcinoma [164], nuclear RZR/ROR receptors were proposed to contribute to the tumor-suppressive effects of melatonin even if, as mentioned above, a direct interaction between melatonin and RZR/RORα receptors is still a matter of debate because no one has yet reproduced Carlberg et al.’s pioneering results that show a direct interaction between melatonin and nuclear receptors (Figure 1).

## 5. Melatonin Antioxidant Properties

Melatonin is an antioxidant, anti-inflammatory and anti-angiogenic molecule. Various oxidative reactions normally occurring in the organism, mainly in mitochondria, generate free radicals from reactive oxygen species (ROS) and reactive nitrogen species (RNS). In normal cells, these species are required for signal transduction before their elimination through endogenous antioxidant compounds and enzymes. The aberrant accumulation of reactive oxidant species can cause multiple lesions in macromolecules (nucleic acids, proteins, and lipids), leading to their damage. In cancer the aberrant activation of pathways leading to cell proliferation and invasiveness causes a hyperaccumulation of ROS and RNS. Endogenous antioxidants are not sufficient to counteract this accumulation. However, cancer cells often acquire resistance to oxidative stress and escape free radical damage. In that context, ROS accumulation in turn promotes tumor development and progression and induces increased cell proliferation, evasion of apoptosis, tissue invasion-metastasis, and angiogenesis (reviewed in [165,166]).

Melatonin counteracts the oxidative stress through multiple mechanisms [167]. It stimulates the expression and activity of antioxidative enzymes [168]. It inhibits the expression of QR2 enzyme at pharmacological concentrations that are higher than those required for MT1 and MT2 activation. Given that QR2 reduces quinones into quinols, and thereby functions as an indirect producer of ROS, it has been proposed that the inhibition of QR2 activity may in part explain the antioxidant properties of melatonin [42].

Moreover, melatonin preserves the integrity and the function of the mitochondria [169,170,171]. Through these mechanisms, melatonin prevents the genotoxic and carcinogenic effects of oxidative stress and helps to maintain cell function and survival.

## 6. Clinical Studies

Melatonin is involved in several physiological processes, and its deficiency (or an altered expression of its receptors) has been associated with a number of chronic diseases including several types of cancer [172,173,174,175,176]. Conversely, a number of randomized and controlled clinical trials showed that exogenously administered melatonin has, among several biological effects, anti-cancer, anti-inflammatory and antioxidant properties in different cancer types, thus improving the responses of patients to traditional therapies and reducing the side effects of the latter [23,24,25].

In the following section, we will present results from observational, translational and cohort prospective studies on the association between pre-diagnostic prolonged exposure to daylight and low melatonin serum levels and subsequent cancer development.

### 6.1. Circadian Disruption and Increased Light Exposure Contribute to Increased Cancer Risk

In 1991 [177], Hann and co-workers showed a reduced risk of breast cancer in blind women. Based on the observation that blind women are constantly in the dark and that melatonin production is increased during the night [178], this study suggested for the first time a possible protective role of melatonin in blind women against the risk of developing cancer. Later observations supported this hypothesis [179,180,181]. Conversely, several bodies of evidence showed that disruption of the circadian rhythm, in part as a consequence of night-shift work and light pollution at night (LAN), increases the risk of developing breast cancer [182,183,184] and prostate cancer [185]. Importantly, our group and others showed an inverse correlation between night work, circadian disruption and melatonin production suggesting a protective role of melatonin against diseases associated with circadian disruption [35].

### 6.2. Low Levels of Endogenous Melatonin or Altered Expression of Its Receptors Are Associated with Increased Cancer Risk

Many groups, including ours, showed that high levels of endogenous melatonin measured many years before the onset of breast cancer were associated with a reduction of breast cancer occurrence [15,16]. Other sets of evidence suggested a protective role of circulating melatonin on prostate cancer development [17].

Conversely, two recent translational studies showed a lower expression of MT1 and MT2 receptors in colon cancer tissues compared to matched normal tissues, suggesting melatonin’s protective role in colon cancer development [159,160]. A negative correlation between melatonin receptor expression and cancer has also been observed in Oral Squamous Cell Carcinoma (OSCC), where a reduced expression of MT1 is also related to the T stage of tumor [186], and in breast cancer, where a lower MT1 expression is associated with a poorer prognosis [156], together with a higher tumor grade and TNM staging [187]. Finally, in Renal Cell Carcinoma (RCC) MT1 receptor expression was found to be lower in cancer tissue compared to normal tissue [104].

## 7. A Possible Crosstalk between Melatonin Signaling and the Hippo Tumor Suppressor Pathway

As mentioned above, melatonin signals in part through MT1 and MT2 GPCR receptors. Recently, GPCR signaling has been shown to regulate the Hippo pathway, which controls animal organ development and growth and whose dysregulation is often involved in tumorigenesis (reviewed in [188]). Components of the Hippo pathway include membrane-associated proteins that sense cell polarity, cell density, and mechanical and metabolic cues that in turn activate a cascade of kinases with adaptor proteins whose final targets are the transcriptional coactivators YAP and TAZ. YAP/TAZ work as oncogenes in many solid cancers, where they are often upregulated or hyperactivated compared to normal tissues (reviewed in [188]).

When the Hippo cascade is on, phosphorylation of YAP and TAZ by LATS1/2 kinases results in their nuclear export, cytoplasmic retention [189,190,191,192,193,194], and degradation by the proteasome [195,196,197]. When the Hippo cascade is off, YAP/TAZ are dephosphorylated and are able to exert their nuclear function and promote transcription of oncogenes in association with oncogenic transcription factors such as TEADs, SMADs, and others [188].

Since YAP/TAZ are becoming increasingly attractive and promising therapeutic targets in cancer treatment (reviewed in [198]), much importance is being placed on the discovery and characterization of inhibitors of YAP/TAZ oncogenic function. What is melatonin’s role in this scenario? At present, no literature has been produced on this topic. However, numerous independent sets of evidence suggest a potential antagonism between melatonin signaling and YAP/TAZ oncogenic function; we will try to summarize them in the following sections.

### 7.1. Gαs May Be a Common Molecular Intermediate between Melatonin Signaling and GPCR/YAP/TAZ Signalng

GPCR signaling regulates YAP/TAZ in response to several biochemical stimuli and YAP/TAZ can be either activated or inhibited depending on which GPCR and subsequent Gα protein is activated. For example, LPA, S1P, and thrombin activate Gαi, Gαq, and Gα12/13, which, in turn, activate YAP/TAZ by inducing their dephosphorylation mediated by Protein Phosphatase 1A (PP1A) and by repressing LATS1/2 kinase activity. This mechanism requires the Rho GTPase RhoA and its associated kinase ROCK and results in YAP/TAZ nuclear translocation [199,200,201,202,203]. In contrast, glucagon, epinephrine, and dobutamine, which transmit signal from Gαs, inhibit YAP/TAZ. One of the proposed mechanisms mediated by Gαs is an increased intracellular cAMP that leads to the activation of protein kinase A (PKA). This in turn inhibits the RhoA/ROCK signaling and stimulates LATS1/2 to phosphorylate YAP/TAZ, which are sequestered in the cytoplasm [203,204,205]. The inhibitory effect of cAMP accumulation on oncogenic YAP/TAZ is conserved in different cell lines, including breast metastatic MDA–MB-231, U2OS, MCF10A, HEK293A, and mouse embryonic fibroblasts (MEFs) [205].

**Figure 2:**
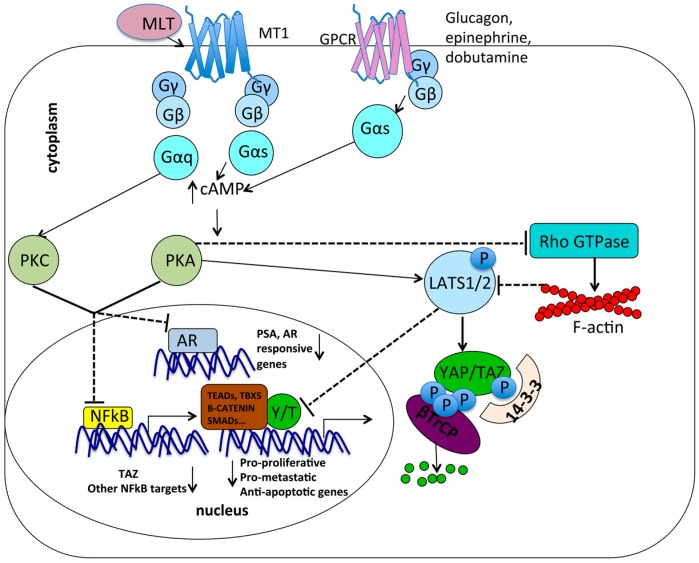
Interplay between G-Protein Coupled Receptors (GPCR) signaling regulated by melatonin and GPCR signaling regulating YAP/TAZ. MT1 binding by melatonin (MLT) induces activation of associated Gαq and Gαs that leads to the accumulation of intracellular cAMP that in turn activates Protein Kinase A (PKA) and PKC. These in turn inhibit NF-κB transcriptional activity on its target promoters, including TAZ promoter. In Androgen Receptor (AR) positive cells, PKA and PKC inhibit the androgen response on AR responsive genes. In parallel, glucagon, epinephrine, and dobutamine signal through Gαs, inducing increased intracellular cAMP and activation of PKA. This in turn inhibits the RhoGTPase RhoA and activates LATS1/2 kinases, resulting in phosphorylation of YAP/TAZ, their cytoplasmic sequestration by 14-3-3 protein, their degradation mediated by βTrCP, and the impairment of their nuclear activity on pro-proliferative, pro-metastatic, and anti-apoptotic genes. ↑ indicates an increase in protein levels or activity; ↓ indicates a decrease in protein levels or activity.

Similarly, melatonin has been shown to activate Gαs proteins associated with MT1 receptors in prostate cell lines [53,54,55,65,66,67], Cos-7 cells [68], and HEK293 cells [69], as well as to increase intracellular cAMP with subsequent activation of PKA and PKC. Thus, the activation of PKA and PKC mediated by Gαs in response to different stimuli (glucagon, epinephrine, dobutamine, melatonin) may lead to inhibition of cell proliferation and invasiveness through multiple converging mechanisms, including LATS1/2 activation [203,204,205] and, as mentioned above, inhibition of NF-κB transcriptional activity and inhibition of the AR response in AR positive cells [55]. Moreover, a recent study showed that TAZ promoter is directly targeted and activated by NF-κB [206] suggesting that melatonin may potentially inhibit YAP/TAZ pro-oncogenic function either through increasing LATS1/2 activity (following PKA and PKC activation) or reducing TAZ transcription (following NF-κB inhibition) (Figure 2).

### 7.2. Metabolic Pathways: Antagonism between Melatonin and YAP/TAZ

Beyond GPCR signaling, YAP/TAZ are also regulated by cell-cell contact, mechanical forces, and metabolic cues. These induce specific intracellular signaling affecting YAP/TAZ function through Hippo kinase cascade-dependent and independent mechanisms. Before going into more detail, some of these mechanisms may crosstalk with melatonin signaling. In general, we hypothesize an antagonism between melatonin and YAP/TAZ on multiple mechanisms involved in tumorigenesis.

Insulin, insulin-like growth factors (IGF-I), nutrient intake, and other growth factors upregulate cellular biosynthetic pathways to sustain cellular growth and proliferation through the activation of protein kinases AKT/PI3K and mammalian target of rapamycin (mTOR) [207,208,209,210,211]. It has recently been shown that insulin and GPCR signaling engage in crosstalk and synergize to positively regulate YAP nuclear function onto YAP/TEAD target genes in pancreatic ductal adenocarcinoma (PDAC) cells via PI3K activation [212]. YAP has been shown in turn to positively regulate the insulin and the IGF-1 signaling [213] to drive IGF-2 expression, activate mTOR signaling and AKT [214,215,216], promote glucose uptake and glycolysis [217], driving growth advantage, metastatic competence, angiogenesis, and therapy resistance in various model systems. On the other hand, melatonin was shown to inhibit AKT/mTOR signaling in models of ovarian cancer [104], breast cancer [218], hepatoma [219], and melanoma [220], where AKT/mTOR are aberrantly hyperactivated and contribute to carcinogenesis [221]. Moreover, melatonin decreases insulin production from pancreatic β cells while increasing the expression and secretion of glucagon from pancreatic α cells [222]. Since glucagon is a negative regulator of YAP/TAZ nuclear function, as mentioned above, melatonin may indirectly inhibit nuclear YAP/TAZ through glucagon upregulation.

Interestingly, AKT and the Insulin Receptor Scaffold 4 (IRS4) have been shown to co-purify with MT2 receptor by Daulat and co-workers [223,224,225].

**Figure 3:**
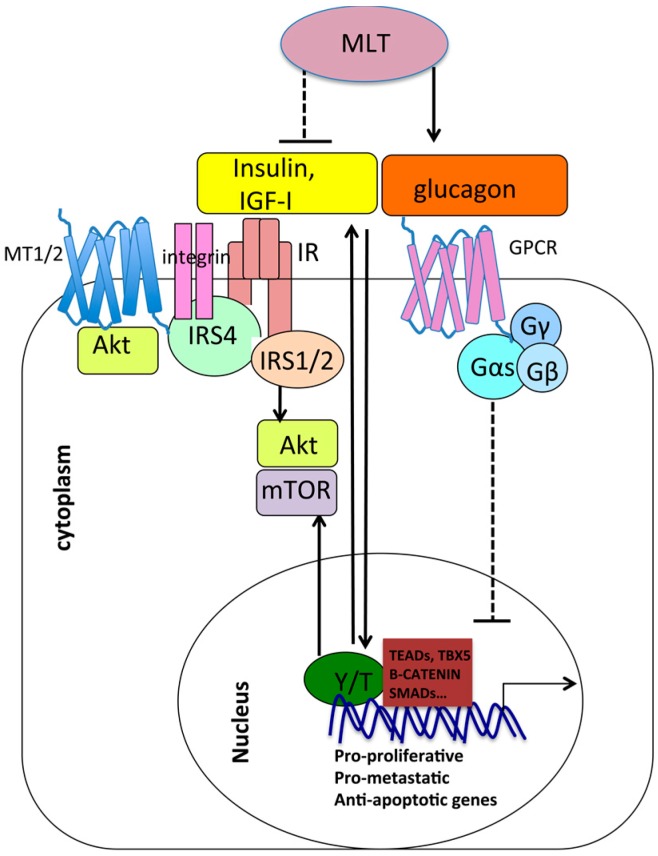
Interplay between melatonin, YAP/TAZ, and metabolic pathways. Melatonin (MLT) upregulates glucagon production and downregulates insulin production and signaling. Glucagon inhibits YAP/TAZ nuclear function through Gαs signaling. Conversely, insulin and GPCR signaling synergize to positively regulate nuclear YAP onto YAP/TEAD target genes. In addition, YAP/TAZ activate AKT and mTOR, which are part of the insulin signaling. In conclusion, melatonin may inhibit YAP/TAZ nuclear function by inducing glucagon expression and decreasing insulin expression. On the other hand, YAP/TAZ positively regulate insulin signaling, and, vice versa, insulin signaling positively regulates YAP, suggesting an antagonism between melatonin function and nuclear YAP/TAZ function. Arrows indicate activation, while dashed and blunt lines indicate inhibition. The figure also shows the interaction between the insulin receptor scaffold 4 (IRS4) with insulin receptor, integrins, and MT1/2 receptors potentially linking these transmembrane proteins at the cell membrane. IR = Insulin Receptor, IRS1/2/4 = insulin receptor scaffold 1/2/4.

Together, this body of evidence suggests potential cross-talk between melatonin signaling, Hippo signaling, and insulin–glucagone signaling, in agreement with a growing literature that showing a reciprocal regulation between YAP/TAZ and metabolism on the one hand [226] and between melatonin and metabolism on the other [227,228] (Figure 3).

### 7.3. Mechanotransduction and Chemoresistance: Opposite Roles of Melatonin and YAP/TAZ

Mechanotransduction is a process where mechanical forces coming from the extracellular matrix (ECM) and from the cytoskeleton are transduced into cellular biochemical signals to regulate cell growth and survival. YAP/TAZ are widely recognized mechanotransducers and mechanoeffectors. They are preferentially active in the nucleus when cells are grown at low density, or on a stiff extracellular substrate, conditions where the cell–ECM contact area is larger and the cytoskeleton is subjected to a stronger mechanical stimulation (often the case of a tumor microenvironment). Conversely, YAP/TAZ effectors translocate to the cytoplasm in response to high cellular density/cell contact, or on a soft extracellular substrate, where the cell experiences lower mechanical stress [189,229,230,231,232,233,234,235,236,237]. Once activated, YAP/TAZ are able to regulate genes involved in extracellular matrix remodelling [238,239]. Matrix rigidity plays an important role in tumor development because it changes during tumorigenesis and regulates cell proliferation, stemness, and invasiveness, and also the response of cancer cells to various chemotherapy agents, through different pathways including YAP and TAZ regulation among others [240,241,242,243]. Accordingly, a role for YAP/TAZ in increasing the resistance of cancer cells to various chemotherapy agents has been extensively documented [243,244,245,246,247,248]. Conversely, melatonin treatment has been shown to partially overcome resistance to chemotherapy, suggesting a possible antagonism between melatonin and YAP/TAZ in cancer chemoresistance [24,102,103,130,131,132,133,134]. In response to mechanical stress, the stabilization of cytoskeletal F-actin fibers and the activation of RhoA–ROCK facilitate YAP/TAZ nuclear translocation, while F-actin destabilization induces YAP/TAZ phosphorylation and cytoplasmic retention. Currently, the gap between YAP/TAZ and these upstream transducers remains to be filled and it would be interesting to see what role melatonin signaling may play in this process. Some bodies of evidence suggest melatonin’s role in mechanotransduction: melatonin has been shown to regulate cytoskeletal dynamics in vitro [249,250,251] and in vivo [252,253], and to reduce the expression of the RhoA kinase ROCK [124,126,127]. Moreover, Daulat and coworkers characterized several proteins interacting with MT1 and MT2, among which is Filamin A [223,224,225], an actin-binding protein that contributes to the cross-linking of cortical actin filaments into a dynamic three-dimensional structure and is involved in mechanotransdution [254].

Akbarzadeh and co-workers’ recent work showed that ovarian cancer cells responded differently to melatonin treatment (in terms of cell proliferation, morphological changes, and stemness) depending on the composition of the extracellular matrix where they were cultured [145]. In this work, the authors showed for the first time the role played by mechanical cues in regulating the response of cells to melatonin.

### 7.4. Cell Contact/Polarity and RhoA/ROCK Signaling

Epithelial tissues line the surface of the animal body and internal cavities. They are composed of cells oriented in the space with an apical-basal polarity. Several proteins contribute to the proper cell-cell adhesion, orientation and spatial organization within the tissue and their dysregulation can promote tumor development and metastasization [255,256,257]. In general, proteins involved in cell contact/junction and cell polarity negatively regulate YAP/TAZ nuclear function by sequestering YAP/TAZ at the apical plasma membrane, thus excluding them from the nucleus, and by interacting with and activating Hippo pathway core kinases [195,255,258,259,260,261,262,263,264,265,266,267,268,269,270,271,272]. Catenin δ1 (p120 catenin), a scaffold protein linking cytoskeletal actin fibers to adherens junctions at the plasma membrane, has been co-purified with MT1 and MT2 by Daulat and co-workers [223,224,225]. p120 has been shown to inhibit nuclear YAP and TAZ when localized at cellular junctions through inhibition of RhoA–ROCK signaling [273,274,275,276], as well as to stabilize cell adhesion cadherin complexes [277,278,279] that negatively regulate YAP/TAZ nuclear function [270,280]. Melatonin by itself reduces the migration and invasiveness of different cancer types by increasing the expression of E-cadherin and other adhesion molecules [122,123,124,125] and by reducing the expression of the RhoA kinase ROCK [124,126,127]. Rac1, a Rho GTPase that can functionally counteract RhoA [281,282], and the Ras-related GTPase Rap1 that activates Rac1 [283], have been co-purified with MT1 [223]. Together, this body of evidence suggests a possible role of melatonin and proteins associated with their receptors in the inhibition of the YAP/TAZ pro-oncogenic and metastatic function, through the inhibition of RhoA-ROCK signaling and via the stabilization of cell surface adhesion proteins [256].

**Figure 4:**
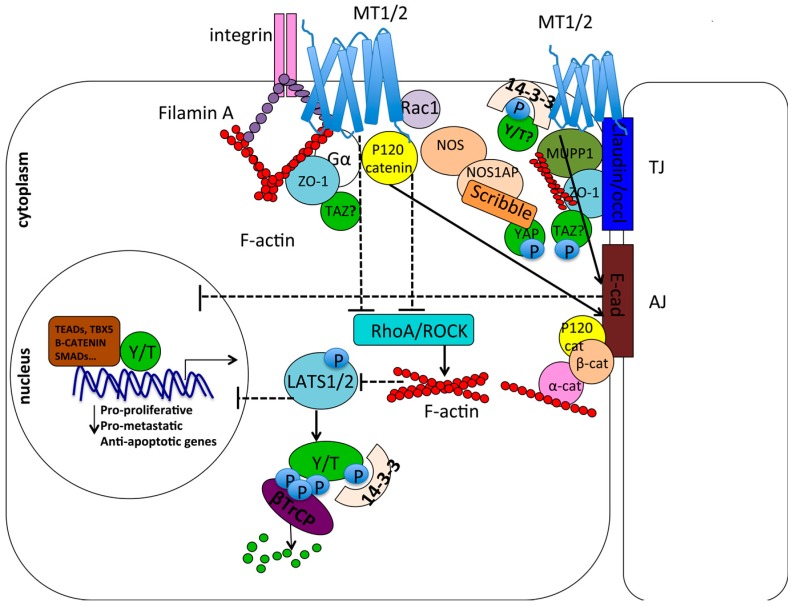
Interplay between melatonin signaling, cell contact-cell polarity complexes, mechanotransduction, and YAP/TAZ. Melatonin signaling inhibits RhoA/ROCK and increases the expression of cell surface adhesion molecules such as E-cadherin. This suggests that it may inhibit YAP/TAZ nuclear function, which in turn is promoted by RhoA/ROCK and inhibited by cell adhesion molecules. P120 catenin has been co-purified with MT1/MT2 receptors. When localized at the plasma membrane, it stabilizes E-cadherin at the adherens junction (AJ) while inhibiting RhoA–ROCK, thus inhibiting nuclear YAP/TAZ (Y/T). MT1/2 also co-purified with MUPP1 scaffold protein, which interacts with ZO-1 at tight junctions (TJ). Moreover, several studies showed that ZO-1 binds Gα proteins. This suggests a possible interaction with TAZ, which has been demonstrated to be sequestered at the plasma membrane through its interaction with ZO-1 at tight junctions. Moreover, YAP/TAZ may be sequestered at the plasma membrane by the 14-3-3 protein, which has been co-purified with MT1 and MT2. MT1/2 have also been co-purified with filaminA, involved in mechanotransdution, suggesting a link between melatonin receptor signaling and mechanotransduction, which has been demonstrated to regulate YAP/TAZ function and to be in turn controlled by YAP/TAZ. Finally, YAP has been co-purified with NOS1AP (nitric oxide synthase1 adaptor protein) in the complex formed with the scribble polarity proteins in proximity to cell–cell contacts. As NOS (nitric oxid syntase) has been co-purified with MT1/MT2, this again may suggest a possible indirect interaction of YAP with MT1/MT2 at the plasma membrane. In general, YAP/TAZ sequestration at the plasma membrane prevents their nuclear pro-proliferative function. Arrows indicate activation, while dashed and blunt lines indicate inhibition.

Moreover, the MT1 receptor has been shown to interact with PDZ domain proteins including MUPP1 and the neuronal NO synthase (NOS) [225,284,285], while MT2 has been co-purified with 14-3-3 protein [223]. MUPP1 is concentrated at tight junctions at the apical membrane and, together with ZO-1 and other scaffold proteins, anchors the integral proteins of tight junctions to the F-actin cytoskeleton and contributes to their correct function and localization [286]. TAZ, containing a PDZ-binding motif, has been shown to interact both with ZO-1 and 14-3-3, which tether TAZ at the plasma membrane, thus inhibiting its nuclear function [189,192,193,268]. Also Gαi2, Gαo, and Gα12 have been shown to interact with ZO-1 in different systems and regulate tight junction assembly and permeability [287,288], while MUPP1 has been shown to promote Gαi coupling and signaling of the MT1 receptor [268,285]. Recently, YAP has been found in complex with the nitric oxide synthase 1 adaptor protein (NOS1AP) at cell-cell contacts together with the Scribble polarity complex [289,290], a negative regulator of YAP/TAZ pro-oncogenic function [245,291]. This interaction increases YAP phosphorylation and cytoplasmic sequestration. Interestingly, MT1 has been co-purified with the nitric oxide synthase (NOS). All these sets of evidence suggest a structural and functional role of cell polarity and cell contact proteins in the regulation of both the Hippo pathway and GPCR/MT1/MT2 signaling, which may converge in the inhibition of YAP/TAZ nuclear function. All these interactions are schematically represented in Figure 4.

### 7.5. Opposite Roles of YAP/TAZ and Melatonin in Androgen–Estrogen Receptor Response and Angiogenesis

Melatonin inhibits the proliferation of Estrogen Receptor α (ERα)-positive lines more efficiently than ERα-negative lines, suggesting that part of its antiproliferative effect is mediated by the inhibition of the estrogen response. In fact, melatonin is able to inhibit the synthesis of steroids as well as interfere with the binding of the ER to its target genes [128,150,151,152,153]. Conversely, other studies showed that melatonin signaling is modulated by antiestrogens in breast and ovarian cancer cells [292]. Similarly, melatonin inhibits Androgen Receptor (AR) response in normal and malignant prostate epithelial cells [147,148,149]. On the other hand, LATS1/2 kinases have been shown to attenuate the androgen response in the prostate by inhibiting AR chromatin binding and transcriptional activity [293] as well as promoting ER degradation and reduction of its transcriptional activity in the breast [294,295], suggesting that melatonin signaling and Hippo signaling may converge to inhibit the ER and AR response.

Finally, melatonin inhibits angiogenesis by interfering with its Hif1α- and STAT3-mediated transcription of VEGF [127,135,136,137,138,139,140,141,142,143,144,145,146]. Conversely, YAP stabilizes H1F1α in response to hypoxia [296], suggesting an antagonistic role of melatonin and YAP/TAZ in angiogenesis regulation.

## 8. Conclusions

At present, promising preclinical and clinical studies suggest that melatonin may be a safe and valid therapy for the treatment of several types of malignancies when administered concomitantly with traditional therapies. In fact, melatonin has been shown to improve the response of patients to different therapies while reducing their toxic effects. On the other hand, preclinical studies showed that inhibitors of YAP/TAZ associated with traditional therapies reduce tumor growth as well as radio- and chemoresistance in different types of cancers [198,215,243,245,246,247,248,297]. To our knowledge, functional crosstalk between melatonin signaling and Hippo/YAP/TAZ signaling has never been previously addressed in the literature. However, several experimental observations may suggest that both the melatonin signaling and Hippo signaling pathways may intersect at different levels (GPCR signaling, AKT/PI3K signaling, and mechanotransduction) and both may potentially inhibit the oncogenic function of YAP and TAZ through many converging mechanisms. Although these potential cross-talks need extensive experimental validation, they may open up a new field of investigation with important implications for (1) a better understanding of melatonin- and YAP/TAZ-mediated pathways, which are still not completely elucidated; and (2) the potential design of novel combinatorial cancer treatments. Today, in fact, the use of pharmacological inhibitors of YAP/TAZ is still in the preclinical phase, while melatonin is used in clinic in combination with other traditional therapies. Studies in this new direction might be worth pursuing.
